# Class Time Physical Activity Programs for Primary School Aged Children at Specialist Schools: A Systematic Mapping Review

**DOI:** 10.3390/ijerph16245140

**Published:** 2019-12-16

**Authors:** Chloe Emonson, Jane McGillivray, Emily J. Kothe, Nicole Rinehart, Nicole Papadopoulos

**Affiliations:** 1 Deakin Child Study Centre, School of Psychology, Faculty of Health, Deakin University, Geelong VIC 3220, Australia; jane.mcgillivray@deakin.edu.au (J.M.); Nicole.rinehart@deakin.edu.au (N.R.); Nicole.papadopoulos@deakin.edu.au (N.P.); 2 Data Science Unit, School of Psychology, Faculty of Health, Deakin University, Geelong VIC 3220, Australia; emily.kothe@deakin.edu.au

**Keywords:** physical activity, special education, class, children, disability, mapping review

## Abstract

Children with disabilities tend to be less active than typically developing peers and may therefore miss important developmental benefits. Class time physical activity (PA) programs can provide additional PA to children and have shown to contribute to numerous benefits in mainstream classrooms. However, it is unclear whether class time PA opportunities are provided in specialist education settings. This review aimed to identify and map class time PA programs that have been implemented in specialist schools and classes. Nine electronic databases were searched. Grey literature searches were also conducted. Programs were included if they were implemented in a primary/elementary specialist school or class, involved a PA component, were conducted during class time and involved more than one child from the class participating. Included programs were mapped and narratively synthesised according to activity type. Of the 2068 records screened, 34 programs were included. Programs involving dance/drama activities (k = 11) were most common and programs involving stretching activities (k = 2) were least frequently implemented. Twenty-three programs had been evaluated, of which only two were randomised controlled trials. More class time PA opportunities are warranted in specialist education settings. Further research is required to build the evidence base for these programs.

## 1. Introduction

Approximately 150 million children worldwide experience a disability [1]. According to the International Classification of Functioning, Disability and Health (ICF) framework, disability is a multidimensional concept that reflects impairments in body structures/function, limitations performing basic activities, and/or restrictions of participation in any area of life [1]. Children with a disability may experience impediments in a range of areas of functioning including sensory and speech, physical, psychological, intellectual and/or other functioning, or may experience impairments arising from head injury, stroke or brain damage [2]. Experiencing a disability can present challenges for a child’s development. Additionally, children with disabilities often experience multiple conditions that co-occur simultaneously and can adversely impact wellbeing [3,4,5]. However, having a disability does not inherently equate to poor health [6]. Protective strategies that are conducive to promoting public health are available to children with disabilities. 

One such protective mechanism is engaging in physical activity (PA) [6,7]. Engaging in PA is related to a multitude of biopsychosocial benefits for children [8,9]. For example, PA has been associated with obesity prevention, physical fitness, motor functioning, self-concept, the development of friendships, improvements in depression, and cognition and sleep benefits [8,9,10,11,12,13,14]. Additionally, participation in group-based organised PA specifically can benefit social functioning for children with developmental disabilities [15]. While guidelines recommend engaging in moderate-to-vigorous intensity PA to achieve health benefits [14,16], emerging research proposes that light intensity activities are also beneficial [17,18]. Indeed, the ICF framework suggests that increased participation in PA could influence a child’s ability to perform basic activities and possibly contribute to changes in body structures/function [19]. Furthermore, engaging in PA may help to reduce the adverse impact of health complexities commonly experienced by individuals with disabilities [20]. This is particularly paramount for children, given the prevalence of illnesses such as obesity reported in the literature [21,22].

A recent review by Jung et al. [7] found that while children with disabilities may engage in a similar amount of light intensity PA as peers without disabilities, they engage in significantly less overall and moderate-to-vigorous PA. Indeed, other literature tends to agree that children with disabilities are less active than their typically developing peers [23,24,25,26] and often fall below the recommended amount of daily PA [16,27,28,29]. Youth with disabilities have also been found to spend most of their time in sedentary behaviours [29,30]. It is important to consider ways to address the lack of engagement in PA for children with disabilities from a public health perspective, as inactivity paired with the health complexities that children with disabilities often experience may put these children at increased risk of experiencing associated illnesses and adversely impact their developmental trajectory [10]. Clearly, programs that support the provision of PA are necessary. 

The large amount of compulsory time that children spend in education settings positions schools as a promising avenue for providing PA opportunities to children [27,31,32]. Schools may be a particularly important setting for increasing PA among children with disabilities given that unlike typically developing children, more of the PA undertaken by children with disabilities occurs during the school day than during out-of-school hours [33]. Promoting PA in primary/elementary schools in particular capitalises on the documented stability of PA patterns established during childhood, which have been shown to persist into adulthood [34]. Promoting PA in primary/elementary schools may therefore help to establish healthful lifelong habits and reduce the negative consequences of inactivity for children with disabilities.

There are numerous opportunities throughout the school day to promote PA, including during physical education (PE), recess periods and even classroom time [32,35]. This is supported by the Comprehensive School Physical Activity Program (CSPAP) framework, which identifies several avenues across the whole school that should be utilised in combination to facilitate students’ PA participation [36]. However, evidence suggests that current PA opportunities provided during non-classroom time in specialist schools (i.e., during PE classes and recess breaks) are not sufficient, as children with disabilities accrue only 14.6% of the total recommended amount of PA for a week during these periods [37]. Indeed, children attending specialist schools have shown to spend about 50% of their time during structured and unstructured PA opportunities (e.g., PE and free play) being sedentary [38], and also spend most of their recess time standing or walking and spend minimal time in sport related activities [27]. This contrasts with mainstream school settings where children only spend 35% of their outdoor break time being sedentary and instead spend 27% of their time in sports [39]. Therefore, children attending specialist schools may benefit from supplementary PA opportunities across the school day [40]. Providing extra PA opportunities during class time could not only maximise PA participation, but also promote wide-reaching associated developmental benefits [31,41]. For example, class time PA opportunities may contribute to psychosocial benefits (e.g., increased self-esteem and social skills) and academic learning benefits (e.g., improved cognitive abilities and classroom behaviour) for children with special needs [35]. 

Classrooms are particularly well suited to facilitate PA programs, given children spend most of the school day in class instructional time [31]. Numerous classroom PA programs such as *Take 10!^®^* and *Energizers* have been implemented in mainstream classrooms around the world [42,43,44,45,46,47]. Some of these programs have involved active breaks, where students engage in self or teacher led games and activities that are separate from academic instruction to interrupt sedentary behaviour [43,44]. Other programs have involved active lessons, where students engage with learning content while doing PA [42,45,46]. However, a review by Naylor, et al. [48] identified numerous factors that may hinder implementation of school-based PA programs and described time to be the most consistently reported barrier to implementation [48]. Time barriers can include preparation and delivery time related to the program, competing curricular demands and teacher overload [31,48]. Unsurprisingly, teachers have reported favouring short (5–10 min) PA breaks that are simple to implement and require minimal preparation and equipment [49]. Therefore, brief active “breaks” specifically may play an important role in supporting the provision of PA during school and should be given distinct consideration when investigating class time PA programs. 

Previous studies have reviewed class time PA programs in mainstream schools and have reported positive effects for children [31,50,51,52,53]. For example, a systematic review of studies that implemented the *Take 10!* program indicates that participation in this program increased children’s PA levels, significantly reduced girls body mass index (BMI) z scores, increased primary school student’s attraction to PA and reduced student’s off-task and inattentive classroom behaviours [50]. Other reviews agree that these programs can contribute to health outcomes including increased PA levels, decreased sedentary time and increased fitness [31,52], and can also contribute to academic outcomes including increased on-task behaviour, enhanced cognitive functioning and improved academic achievements [31,51,53].

Given this success, it is possible that class time PA programs may have a positive impact on the development of children with disabilities attending specialist schools [35]. However, while there are published reviews of general interventions to increase PA participation for children with disabilities [54,55,56] and mainstream classroom-based PA interventions [50,51], to the author’s knowledge, no review of class time PA programs implemented in specialist primary schools currently exists. It is therefore unclear whether children who attend specialist schools have opportunities to participate in class time PA programs like their typically developing counterparts. It is also not possible to know whether these programs contribute to positive health outcomes for children attending specialist schools. This knowledge is critical for navigating the future development and investigation of class time PA programs in specialist education settings. 

The aim of this study is to conduct a systematic mapping review of class time PA programs that have been implemented in specialist primary schools and classes. Specifically, this review will identify what the programs have involved, what outcome variables have been evaluated after implementation and whether any programs are conducted specifically as a short break. This review will also comment on the ability of class time PA programs to produce positive outcomes (including physical, behavioural, academic, cognitive, psychological, social and “other” outcomes) for children with disabilities.

## 2. Materials and Methods 

This systematic mapping review was conducted from September 2018 to June 2019. A protocol was registered with the Open Science Framework on 24th July 2018 (http://osf.io/q7smv/). 

### 2.1. Eligibility Criteria

#### 2.1.1. Population

Programs implemented with primary/elementary students attending a specialist school or class were eligible. This could involve children with a variety of disabilities, including but not limited to physical (e.g., cerebral palsy), intellectual (e.g., Down syndrome), psychological (e.g., autism spectrum disorder; ASD), sensory (e.g., hearing impairments), multiple, or other disabilities and special needs (e.g., learning difficulties). Programs in inclusive or mainstream classes were excluded. Where school or grade level was not clear, programs were eligible if participants mean age was between 5 and 13 years. Programs implemented with middle and secondary school students specifically were excluded. Programs implemented with both primary/elementary and secondary students were eligible if results for each group were reported separately; however, only results from the primary/elementary group were considered. 

#### 2.1.2. Intervention

Programs that contained a PA component of any intensity and duration that were conducted during general class instructional time in the school day and involved two or more children from the same class participating were considered for inclusion. PA in this review refers to any bodily movement that results in some energy expenditure [57] including games and play, not limited to moderate-to-high intensity activities. Programs conducted outside the classroom (e.g., in a gymnasium) were eligible, providing the program was conducted during class time. Additionally, programs that included a PA component among other activities (e.g., academic tasks) were eligible, however, only information about the PA segment of multi-component programs was utilised for mapping purposes. Programs that involved children participating individually were excluded. PE programs were also excluded, as this review aimed to identify supplementary class time programs. 

#### 2.1.3. Comparison

Studies with and without a comparison were eligible for inclusion.

#### 2.1.4. Outcome

Studies that evaluated any type of outcome after program implementation were considered. Studies without outcome evaluation of the program were also eligible. 

#### 2.1.5. Study Type

Documents using any design to describe the implementation of a class time PA program were eligible. Peer-reviewed and grey literature documents were considered. No restrictions were placed on date. 

### 2.2. Information Sources

Eight electronic databases (MEDLINE Complete, PsycINFO, CINAHL Complete, Academic Search Complete, SPORTDiscus with Full Text, Global Health, Education Source and ERIC) were searched via EBSCOhost to identify relevant studies. Embase was also searched. Literature was searched from the earliest date in each database to 7th September 2018. 

Grey literature was searched during September and October 2018 using ProQuest Dissertations & Theses Global and Google to identify additional programs. The first 50 results from 28 Google searches of Australian health sites, Australian state government and education sites, United States of America (USA), United Kingdom (UK), Canada, Japan, New Zealand, Singapore and South Africa federal education sites, United Nations Children’s Fund (UNICEF), United Nations Educational, Scientific and Cultural Organisation (UNESCO) and World Health Organisation (WHO) sites were reviewed. See Supplementary Table S1 for a list of the Google searches conducted. Reference lists of all included results were hand-searched to identify additional records. 

### 2.3. Search Strategy

The search strategies were developed with the support of an experienced librarian. For the electronic databases, truncation was applied to key terms (see Table 1) which were searched in titles and abstracts. Subject headings corresponding to the key terms were also sourced from each database (see Supplementary Table S2 for an example). The five concepts were then combined using the “AND” boolean operator. All database searches were limited to the English language as this review did not have resources for a translator. 

The Google search strategies included a series of key terms with the relevant Google operator to limit each search to one of the sites described above (see Supplementary File S3 for an example). The ProQuest Dissertations & Theses Global search strategy used the concepts displayed in Table 1 combined using the “AND” function. The key terms in each concept were searched “Anywhere except full text-NOFT” and the search was limited to English language. 

### 2.4. Study Selection

Each electronic database was searched individually (CE). De-duplicated results were imported into Rayyan for dual eligibility screening (CE and NP), first by title and abstract. Remaining results were reviewed in full-text independently by two authors (CE and NP). The same process was followed for the ProQuest Dissertations & Theses Global search. Two authors (CE and NP) conducted the Google searches and reviewed the first 50 results of each search simultaneously. The authors met to resolve any discrepancies throughout the entire selection process. If more information was required to determine the study’s eligibility, an attempt was made to email the author if contact details could be obtained. This was not always possible due to the age of the study. Studies were excluded if the author didn’t respond within one month. 

### 2.5. Data Extraction

Data was extracted by one author (CE) according to a pre-planned form which included bibliographic information, study aims, study characteristics, sample characteristics, intervention characteristics, outcomes and results. A second author (NP) verified initial data extraction. 

### 2.6. Risk of Bias

To assess the risk of bias, the Effective Public Health Practice Project (EPHPP) tool [58] was used. The EPHPP tool was applied by two authors (CE and EK) independently to any included study that evaluated the PA program and the authors met to resolve discrepancies. Each study was examined for risk of bias at the individual domain level and also given a global risk of bias score. 

### 2.7. Data Analysis

Findings are presented in tables, maps, and are also synthesised qualitatively according to the type of activities in the class time PA program to describe this field of literature. Maps were generated using RStudio (Version 1.1.463; RStudio, Inc., Boston, Massachusetts, USA). Broad activity categories (dance/drama, games/play, motor activities, running, stretching, swimming and “other”) were formed by authors after final program inclusions were decided to synthesise and map the programs. Variables that had been previously evaluated in the included studies were categorised by authors into broad outcome groups (academic, behavioural, cognitive, physical, psychological, social, or “other” outcomes) for mapping purposes. Variables were mapped according to outcome group per study; therefore, studies that evaluated multiple variables of the same outcome type were categorised once per program. Evaluations of included programs were not considered if results weren’t related specifically to the PA component of the program. Given this review primarily aims to identify programs that have been implemented and a large amount of heterogeneity was found between intervention characteristics and outcomes, it was not appropriate to conduct a meta-analysis. 

## 3. Results

### 3.1. Study Characteristics

**Figure 1:**
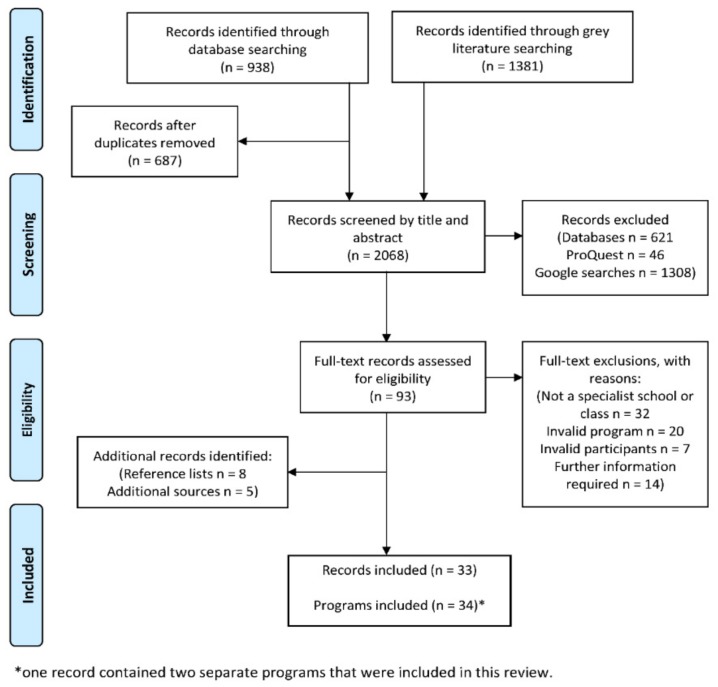
Flow diagram of the review process [63].

**Figure 2:**
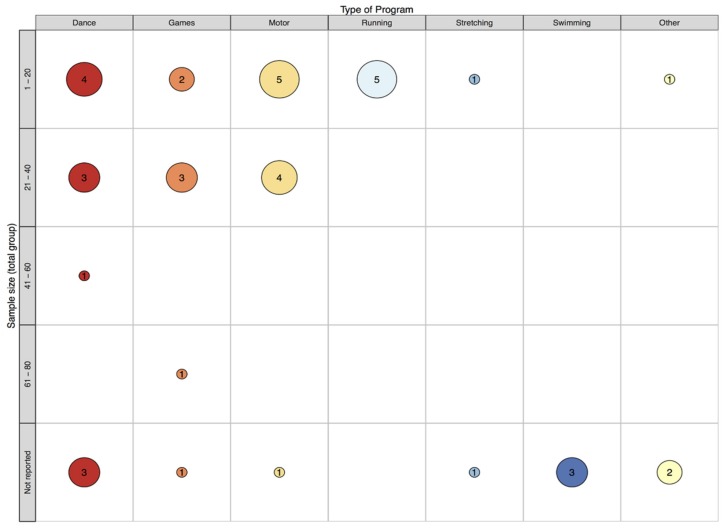
Map of program type and sample size.

**Figure 3:**
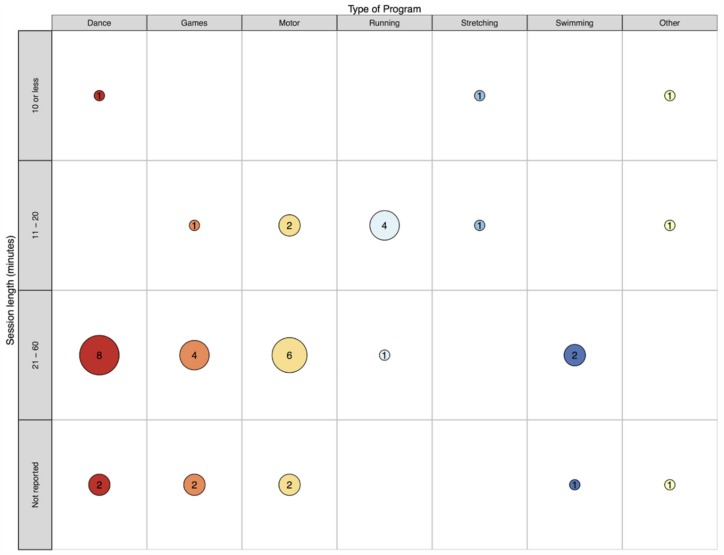
Map of program type and session length.

**Figure 4:**
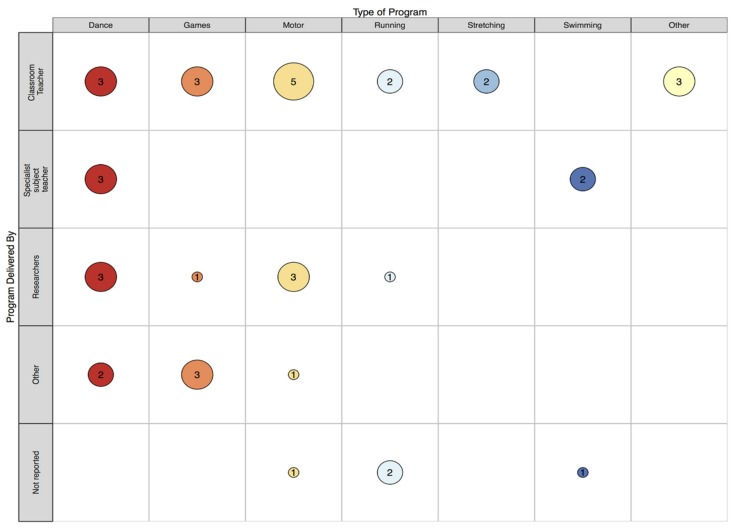
Map of program type and person responsible for program delivery. Note: One person responsible for program delivery was mapped for each record (where reported), however some programs were implemented collaboratively. Refer to Table 3 for a more detailed description of who was involved with implementing each program.

This review identified 33 records that report on 34 class time PA programs (see Figure 1) that have been implemented in specialist schools and classes between 1964 and 2018. More than half (67%) were conducted in the USA (k = 23). This may be unsurprising, given the relatively high prevalence of disabilities in children aged 0–14 years (8.5%) reported in the USA [59]. However, accurately comparing childhood disability estimates across countries is impossible due to methodological and definition differences [59]. The majority of records that reported sample size included a small sample, ranging from four to 67 (see Figure 2). Perhaps due to a higher prevalence of some disabilities (e.g., autism) in boys compared to girls [60], many more boys were involved than girls (289 boys vs. 102 girls) according to the studies that reported participant’s gender. Where reported, children ranged from 3 to 16 years of age. This relatively wide age span is not surprising, as special education classes often consist of students of varying ages [61,62]. The lowest mean age was 5.2 years and the highest was 12.5 years. Programs have most commonly been implemented in classes for children with intellectual disability (ID; k = 14), and least frequently implemented with children with emotional difficulties (k = 1). See Figure 3 and Figure 4 for an overview of program implementation characteristics. Table 2 summarises the characteristics of included records.

**Figure 5:**
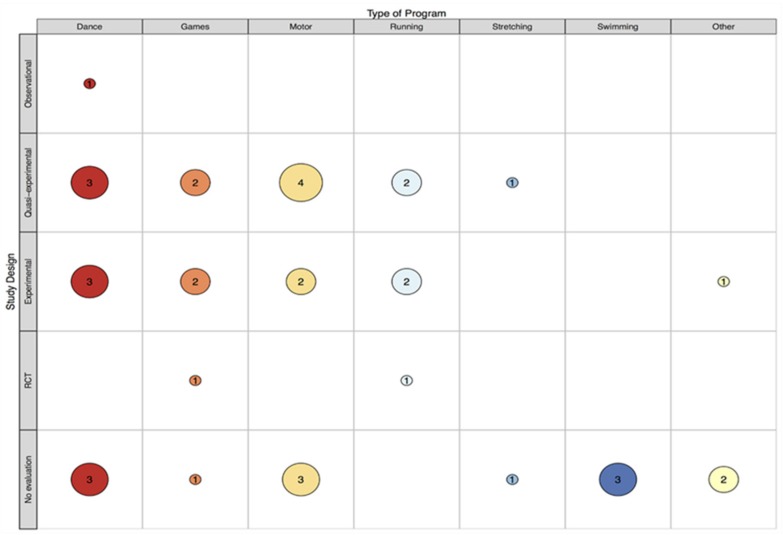
Map of program type and evaluation study design.

**Figure 6:**
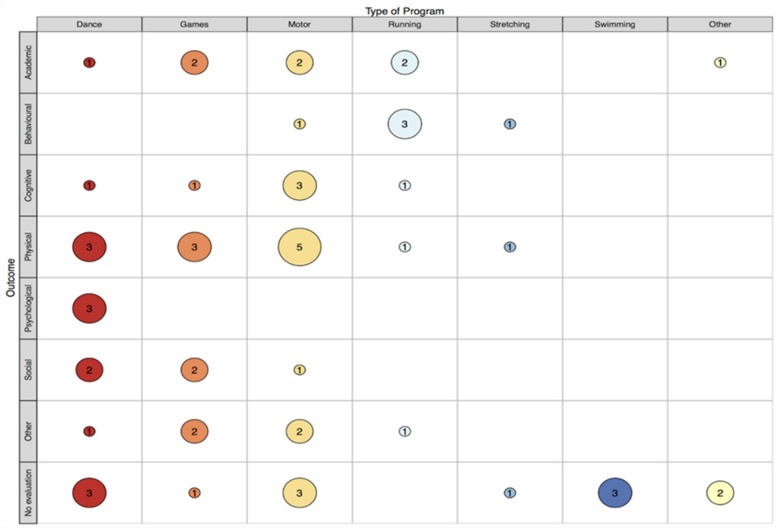
Map of program type and category of outcome evaluated.

Twenty-three programs included in this review had been previously evaluated; however, three of the evaluations [62,64,65] were not specific to the PA component of the program. Although these programs are described in the results section of this review to document PA that has been implemented during class time, the evaluations of these studies [62,64,65] are not considered. Only two evaluations were conducted using a randomised controlled trial (RCT) design (see Figure 5). Overall, physical outcomes were most commonly evaluated. This was followed by academic, behavioural, cognitive and “other” outcomes (e.g., developmental age and family functioning). Social outcomes were less frequently evaluated and psychological outcomes were least frequently evaluated (see Figure 6). 

### 3.2. What PA Programs Have Been Implemented in Specialist Schools and Classes, and What Have They Involved? 

Programs were subjectively categorised into broad activity groups to synthesise the type of activities involved. Some programs involved more than one type of activity and are therefore included in more than one category in the text described below and in the map figures. Refer to Table 3 for a description of the 34 programs included in this review.

#### 3.2.1. Dance/Drama

Class time PA programs most commonly included dance/drama-based tasks (k = 11), which involved activities such as theatre games, aerobic dance routines and acting exercises. These programs have been published since the 1960s and all were conducted in the USA. These programs have been least frequently implemented in classes of children with ASD (k = 1) and multiple disabilities (k = 1), and most frequently implemented in classes of children with ID (k = 4). Programs involving dance/drama activities were often implemented with small samples, with only one containing a total of more than 40 participants.

Programs involving dance/drama activities were commonly implemented by a specialist subject teacher (i.e., a dance teacher; k = 3), researchers (k = 3) and classroom teachers (k = 3). Where location of implementation was reported, programs were slightly more frequently implemented in the classroom (k = 2) and school gymnasium (k = 2) than other school rooms (k = 1) or mixed locations (e.g., a combination of the classroom and gymnasium; k = 1). The shortest program was implemented for two weeks (k = 1) and the longest went for the entire school year (k = 2). Most involved 40–60 min sessions (k = 7), with only one program consisting of ≤10 min sessions. Three programs were implemented once per week, two were twice per week, one was three times per week and two were every school day. 

Seven programs involving dance/drama activities were evaluated, none of which were conducted as an RCT. A variety of outcomes were evaluated including academic (k = 1), physical (k = 3), cognitive (k = 1), psychological (k = 3), social (k = 2) and “other” outcomes (e.g., auditory discrimination abilities; k = 1). Results suggest that programs involving dance/drama activities may contribute to improvements in children’s physical fitness, psycholinguistic skills, visual perception skills and increased positive social interactions with typically developing peers. 

However, the overall evidence produced by these evaluations was consistently weak. Six studies demonstrated risk of selection bias by either consisting of a sample that is not likely to be representative of the target population (k = 5) or providing insufficient information to judge representativeness (k = 1). Only one used a strong study design. Additionally, risk of performance and detection bias is present, as blinding was either not used or reported in any evaluations. Nevertheless, valid and reliable data collection tools were used in some cases (k = 4) and most (k = 6) used appropriate statistical analyses. Studies also controlled for confounding variables where applicable (k = 3).

#### 3.2.2. Motor Activities

Class time PA programs also commonly consisted of motor activities (k = 10), which involved a range of activities including balance, body awareness, locomotor and non-locomotor tasks. These programs were most commonly implemented in the USA (k = 6), with only one program each being implemented in Australia, Canada, South Africa and the UK. Two programs were published in the 1960s. Since then, there have been two programs each in the 1970s and 1980s, and four programs since 2010. Programs involving motor activities have been much more frequently implemented with classes of children with ID (k = 7) than sensory impairments (k = 2) or other special needs (k = 1). Sample sizes were generally small, with all studies involving ≤ 40 participants. 

Where the person who delivered the program was reported (k = 9), most were implemented by classroom teachers (k = 5) or researchers (k = 3). Of the six programs that reported location, three were delivered in the classroom, two in a different school room and one in the gymnasium. Program durations were dispersed, ranging from a minimum of eight weeks (k = 1) to a maximum of 15 months (k = 1). Sessions were generally quite lengthy, with most programs (k = 6) consisting of 30–45 min sessions and only two of ≤ 20 min duration. The majority involved regular sessions, with six programs involving sessions every school day. 

Six of the programs involving motor activities were evaluated; two used an experimental and four used a quasi-experimental design. Physical outcomes were most frequently evaluated (k = 5). Results suggest that class time programs involving motor activities may contribute to a range of positive effects including improved academic abilities, improved gross motor skills (e.g., balance, agility and skipping) and increased cardiovascular and muscular endurance. 

Although four of the six evaluations produced an overall weak evidence rating, there were many strengths. All evaluations used a moderate or strong study design. Furthermore, most studies (k = 4) reported a high participant retention rate and used appropriate statistical analyses (k = 4). Additionally, where applicable, studies either had no differences between groups (k = 2) or attempted to control for relevant confounders (k = 1). However, only half (k = 3) used valid and reliable data collection instruments, and most (k = 5) contained samples that were not likely to be representative of the target population. Additionally, many studies (k = 5) either didn’t report or utilise blinding. 

#### 3.2.3. Games/Play

Other class time PA programs included games/play activities (k = 7), which involved activities such as interactive electronic games, imaginative games (e.g., pretending to be animals), the Hokey Pokey, Simon Says and tag. These programs were most frequently implemented in the USA (k = 3) and most commonly implemented since 2010 (k = 3). They have been delivered in classes of children with ASD (k = 3) and ID (k = 4), and generally involved modest sample sizes, with only one study containing more than 30 participants. 

Programs that included games/play activities were most frequently delivered by the classroom teacher (k = 3) or “other” personnel (e.g., non-specified school staff or art therapists; k = 3). Locations varied, with two programs being implemented in classrooms, one in the school gym, one in mixed locations, and one in a different school room (i.e., the room where PE would normally take place). These programs ranged in duration from eight weeks to the whole school year. Session frequency varied between weekly and daily. One program involved 15 min sessions, whereas four involved 30–45 min sessions. 

Five of the programs that involved games/play activities were evaluated, only one of which was an RCT. Physical outcomes were most frequently evaluated (k = 3) and cognitive outcomes were least frequently evaluated (k = 1). Results suggest that programs containing games/play activities may positively influence children’s academic abilities, social functioning, arm strength and flexibility. 

While three overall rating scores indicate weak evidence for programs involving games/play activities, the studies included a number of domain-level strengths. All evaluations used a moderate or strong study design and most (k = 4) used appropriate statistical methods. Additionally, majority of studies (k = 4) used data collection tools that were both valid and reliable, and had few participants withdraw or drop out (k = 3). Evaluations that recruit more representative samples and use/report blinding processes may increase the quality of evidence.

#### 3.2.4. Running

Multiple running programs (k = 5) have be implemented during class time in specialist schools and classes, all of which were in the USA. Most (k = 3) were implemented in the 1980s, with only one being implemented in the 2000s and one since 2010. Two studies involved children with ASD, one involved children with ID, and two involved children with other special needs. All involved few participants, with N = 12 being the largest sample size. 

Three programs reported who delivered the program, of which, either classroom teachers (k = 2) or researchers (k = 1) were responsible. Unsurprisingly, no running programs were conducted in the classroom. Those that described location were either conducted in school grounds (e.g., around the school playground; k = 2), the gymnasium (k = 1) or a location categorised as “other” (i.e., a quarter mile track; k = 1). It is unknown whether this track was on school grounds or external. Duration of the running programs varied from as little as three weeks to as much as seven months. Majority (k = 4) were conducted for between three and six weeks. Sessions ranged from 12 to 45 min. Where reported, session frequency ranged from twice per week to every school day. 

All running programs were evaluated, one of which was conducted as an RCT. Behavioural outcomes were most frequently evaluated (k = 3). Evidence suggests that running programs may have positive consequences including increased correct academic responses, increased academic engagement, greater attention span, improved classroom behaviour and greater fitness. 

However, the majority (k = 4) of the evaluations produced weak overall evidence. Evidence may be at risk of detection and performance bias due to infrequent use or reporting of blinding of outcome assessors and participants. Only one study reported blinding of outcome assessors. Selection bias may also be present, as studies frequently investigated samples that are not likely to represent the target population. Only one study contained a sample that was somewhat likely to be representative. While three studies employed a moderate or strong study design and two utilised valid and reliable data collection methods, improvements in these domains may strengthen the quality of evidence. Encouragingly, the evaluations generally had few participant withdrawals and drop-outs, and used appropriate statistical methods. 

#### 3.2.5. Swimming

Swimming programs appear to have been less frequently (k = 3) implemented during class time. All three were implemented in Australia, one of which was implemented in 2011. The other two records did not report when the swimming program was implemented but indicated that it was part of the ongoing curriculum. These programs have involved children with ASD (k = 1), ID (k = 1) and children with both ASD and ID (k = 1). While none of the records reported exactly how many children participated, each indicated that all children at the school were involved.

One swimming program did not report who delivered the program. The remaining were delivered by specialist subject teachers, specifically and unsurprisingly, swimming teachers. While it is sensible to assume that each of these programs were conducted at a swimming pool, none explicitly stated whether they were conducted in a pool at the school or an external facility. One program involved lessons every school day for a two-week period. The other two involved weekly lessons for the entire school year. Session lengths differed, with one program not reporting an exact duration but indicating that lessons lasted from between 15 min to 1 h, another program involving 30 min sessions and the third program involving 45 min sessions.

None of the swimming programs identified had been evaluated, therefore, it is unclear whether class time swimming programs contribute to positive outcomes.

#### 3.2.6. Stretching

Class time PA programs in specialist schools and classes were least frequently based around stretching activities (k = 2). Programs that did primarily involve stretching activities included activities such as yoga, slow-motion movements, and tensing, flexing and releasing the body. Both programs were implemented in the USA, one in the 1970s and the other in the 2000s. One was implemented with children with emotional difficulties and the other involved children with multiple disabilities. Only one program reported sample size, which was very small (N = 5).

Both programs were implemented by classroom teachers and delivered in the classroom. One program lasted 16 weeks, while the other did not report total duration. Additionally, one study involved sessions three times per week, while the other study did not report session frequency. Both programs contained brief sessions, with one involving 15 min sessions and the other consisting of no more than 10 min of stretching.

Only one of the programs involving stretching activities was evaluated. Using a quasi-experimental design, this study evaluated the effect of the program on behavioural and physical outcomes. There was a significant reduction in children’s muscle tension following the program. Parents and teachers also noted a reduction in children’s inappropriate behaviours; however this was not statistically significant.

The overall evidence from this evaluation is weak and possibly affected by selection bias, as the sample was not likely to represent the target population. The quality of evidence may also be impacted by the lack of reporting of the reliability and validity of data collection tools and blinding of outcome assessors and participants. This study did however utilise a moderate strength study design, appropriate statistical methods and was unlikely to be impacted by attrition bias, as there were no withdrawals or drop-outs.

#### 3.2.7. Other Activities

Three programs involved activities that did not fit the above categories. These include a TaeBo exercise routine (i.e., martial arts activities), an outdoor education program (e.g., involving walking excursions) and a fitness program involving walking activities. One was implemented in Australia, one in Singapore and one in the USA. Two programs have been implemented since 2010 and one did not report the year of implementation. These programs were implemented with children with ASD (k = 1) and ID (k = 2). Sample size was rarely reported, however, one study reported that six children participated in the TaeBo activity.

All of these programs were delivered by classroom teachers, however, location varied. One did not report where the program was implemented, one was in a school hall and one was in mixed locations (i.e., the classroom and outdoors). Programs ranged in duration from 30 days (k = 1) to the whole school year (k = 1). One did not report total duration. One program included 19 sessions over a 30 day period, while the other two were implemented weekly. Sessions were generally brief, with one program involving 10 min sessions and one involving 15–20 min sessions. One did not report the length of individual sessions.

Only one of these programs had been evaluated. Using an experimental design, the study evaluated the impact of TaeBo activities on academic outcomes and found that performance on mathematics and language tasks improved on most of the days that children engaged in the program.

However, the overall quality of evidence is weak. While statistical methods were appropriate and the study employed a moderate strength study design, the evidence is subject to selection, performance, detection, and attrition bias. Indeed, the sample was not likely to be representative of the target population, outcome assessors were not blinded to intervention status, and there was a lack of reporting regarding blinding of participants and withdrawals and drop-outs.

### 3.3. Types of Programs Implemented with Particular Disability Populations

Disability categories (i.e., ASD, ID, emotional difficulties, sensory impairments, multiple disabilities and other special needs) were formed after record inclusions were finalised in order to synthesise the data. One disability category was assigned per record based on the participants and type of specialist class described. Refer to Table 2 for a description of the participants’ disability and the disability category assigned.

For classes of children with ASD, programs involving games/play activities were most common (k = 3) and demonstrated potential benefit for boys social functioning. This was followed by running programs (k = 2) which may contribute to increased correct academic responses and academic engagement. Programs involving dance/drama, swimming and ‘other’ activities were less common (k = 1 each) and were not adequately evaluated to understand benefits for children.

For classes of children with ID, programs most commonly involved motor activities (k = 7), with some studies indicating that these activities may contribute to improvements in motor abilities, endurance and academic abilities. This was followed by programs involving dance/drama activities (k = 4), with studies suggesting improvements in physical fitness and dance skills, and programs involving games/play activities (k = 4) which may contribute to improvements in gross motor skills and flexibility. Programs involving running, swimming and ‘other’ activities were less common in this population. Nevertheless, studies indicated that running activities may contribute to increased fitness, and ‘other’ activities (i.e., TaeBo) may contribute to improved academic performance.

Stretching was the only activity type used in a class of children with emotional difficulties. Results indicated that stretching activities (e.g., slow-motion movements and tensing/releasing the body) may reduce muscle tension in children with emotional difficulties.

For classes of children with sensory impairments (e.g., blindness or deafness), two programs involved motor activities, with one study suggesting that these activities may contribute to improvements in locomotor development, performance development and practical reasoning development. Two programs also involved dance/drama activities; however these were not evaluated.

Programs for children with multiple disabilities (e.g., severe physical, cognitive and emotional disabilities) have involved swimming (k = 1), stretching (k = 1) and dance/drama activities (k = 1). However, none of these were evaluated to understand the benefits of the program.

For classes of children with other special needs (e.g., learning disabilities), programs have most commonly involved dance/drama activities (k = 3), with one study suggesting that the activities may contribute to improvements in psycholinguistic and visual perception skills and another indicating a reduction in anti-social actions after participation. This was followed by running programs (k = 2), with studies indicating improvements in attention span and classroom behaviour, and a program involving motor activities (k = 1) which was not evaluated.

## 4. Discussion

Class time presents a promising opportunity in which to provide PA for children with disabilities. However, it is not currently clear whether children who attend specialist education settings have opportunities to participate in class time PA and whether these programs contribute to health benefits. This review aimed to systematically search for and map class time PA programs that have been implemented in specialist primary schools and classes. The results serve to identify avenues for further development and investigation of class time PA programs in school settings for children with disabilities.

This is the first review to the authors’ knowledge that explores class time PA programs that have been implemented specifically in specialist schools and classes. Results identified few (n = 34) class time PA programs, which aligns with existing literature suggesting that more active opportunities are needed in specialist school settings [27,35,37]. Notably, reported dropout rates were often low and some studies [71,81] commented that staff and students engaged well with the program, suggesting these programs may be feasible. Nevertheless, a number of factors such as the heterogeneity of students [97], children’s apprehension towards new activities, and safety concerns [35] may pose difficulties for delivering class time PA programs in special education. It is also important to acknowledge the possibility of other class time PA programs being implemented that are not documented in peer reviewed or grey literature. Therefore, readers are cautioned that results and recommendations of this review are based solely on the evidence produced through the described searches. Among the 34 included programs, a range of activities were identified which were grouped into broad categories of dance/drama (n = 11), motor activities (n = 10), games/play (n = 7), running (n = 5), swimming (n = 3), stretching (n = 2) and “other” activities (n = 3). This suggests that a variety of PA may be suitable for implementation during class time in specialist schools.

### 4.1. Program Components

According to activity categorisations in this review, the identified class time PA programs most commonly included dance/drama activities. This is reasonable given research suggests that dance can be used to meet the diverse needs of learners in the school setting [98] and that children with disabilities enjoy dancing [99]. Additionally, a recent review by May et al. [100] indicates that dance programs can have a number of benefits for the development of children with disabilities including physical, cognitive, psychological and social benefits. Therefore, it is perhaps unsurprising that dance/drama activities were common. Moreover, the identified programs involving dance/drama activities were implemented with children with a range of disabilities suggesting that these programs seldom preclude any child with special needs and may therefore be particularly conducive to specialist education. Interestingly, all programs involving dance/drama activities were conducted in the USA. These programs generally involved rather lengthy sessions (n = 8 lasting 21–60 min) which may not be feasible for classroom contexts worldwide due to increasingly demanding time constraints placed upon teachers [48].

It is also interesting to note that all swimming programs identified were implemented in Australia. This is perhaps unsurprising, given that swimming is part of the school curriculum in a number of Australian states [101,102,103]. Arranging the logistics of a class time swimming program may be challenging due to requiring appropriate facilities and qualified personnel to conduct the program. Despite this, swimming should be a relevant consideration for specialist schools in other countries, as research suggests that swimming is a popular choice of PA for children with disabilities [104].

This review identified few programs based primarily around stretching. It is possible that stretching activities may not be a first choice of PA if teachers decide to dedicate class time to maximising children’s energy expenditure. Additionally, characteristics such as poor attention are common among some children with disabilities (including those with Autism, Attention-deficit/hyperactivity disorder and ID) [29,105], which may make stretching activities challenging, particularly with regards to remaining relatively still and sustaining stretching movements. 

Similarly, few running programs have been implemented in recent years, with the latest program being implemented in 2011. This could reflect support for a recent shift towards providing children with more play-based PA opportunities to achieve holistic benefits [106]. The decline in running-based programs may actually be a promising finding, as previous literature reports that children with disabilities tend to dislike running, but will become more interested if it is incorporated into a fun game [104].

Encouragingly, one-third of all programs were implemented within the last decade, and four (11%) in the last five years. This could be in response to calls for action to increase the amount of PA children engage in [16]. Additionally, these programs may have received extra attention in the face of increases in childhood obesity [107]. However, very few programs were implemented with the aim of targeting children’s PA levels or outcomes such as BMI. Given that children with disabilities often fall below the recommended amount of PA and rely on opportunities provided in school to accrue PA [27,33], more research is needed to explore whether class time programs in the specialist school context can increase children’s PA levels and associated health outcomes.

Interestingly, none of the programs in this review utilised existing programs (e.g., *Take 10!* or *Energizers*) that have been successful in mainstream schools. This is despite the features of these existing programs likely being attractive to teachers in that they are often brief, require minimal training and equipment to implement, and can incorporate learning materials [31]. Special education teachers may be apprehensive about whether these programs are suitable for use in their classrooms and it is possible that the programs require adaptation. Fortunately, a study by McMinn, Rowe and Trim [35] reveals agreement among experts regarding the potential for beneficial use of these programs with children with special needs. Future research could consider applying these programs to the specialist school context with adaptations as necessary and evaluating whether they can have similar success.

### 4.2. Classroom “Break” Programs

Only 11 programs included in this review consisted of short sessions (i.e., ≤ 20 min), of which, seven were conducted by classroom teachers. Time and implementation variables may have a large influence over whether teachers include PA during class time. Indeed, classroom teachers are increasingly under pressure to manage competing demands and therefore may be inclined to forfeit time for PA during instructional sessions if they are not invested or supported appropriately [31,32,48]. Future developments should consider designing programs as brief “breaks” that can be easily implemented by specialist education classroom teachers in order to increase the likelihood of sustainable PA being included during class time. Indeed, research suggests that even small amounts of PA can meaningfully contribute to the accrual of PA and health outcomes [8,108]. Additionally, results from studies included in this review that did consist of short sessions demonstrate potential for producing positive outcomes including fewer classroom disruptions, increased academic engagement, improved academic performance and improved social functioning [66,75,77,85]. Therefore, class time PA programs designed as brief breaks may provide a convenient and efficient method of supporting developmental benefits for students with disabilities. 

### 4.3. Program Evaluations

Physical outcomes were most frequently assessed by studies included in this review. Variables categorised as behavioural, cognitive and academic outcomes were also reasonably frequently evaluated. This is perhaps unsurprising, given these variables are proximal to the setting and type of program reviewed. That is, given class time programs implemented in schools were reviewed, it is unsurprising that academic and cognitive outcomes were targeted as these may reflect enhanced classroom performance—a valued result in schools. Similarly, behavioural outcomes may be frequently targeted in class time programs as problematic behaviour is less conducive to a supportive learning environment. Additionally, given PA programs were reviewed, it is unsurprising that many studies targeted physical outcomes. However, as aforementioned, no studies evaluated the impact of the program on children’s PA levels. This may suggest that PA programs in specialist education are targeted more towards advancing aspects of child development (e.g., motor, cognitive and academic skills) than fostering increased PA engagement. Notably, this review identified a lack of studies investigating the impact of class time PA programs on psychological outcomes. Given children with disabilities have shown to experience higher rates of psychopathology [109] and previous literature suggests relationships between PA and psychological wellbeing [11,110,111], more research is needed to understand whether class time PA programs in specialist schools can contribute to positive psychological outcomes for children with disabilities.

Results suggest that class time PA programs may have the potential to yield a range of positive outcomes for children with disabilities. For example, there are indications of improvements in social functioning, classroom behaviour, academic performance, physical fitness, cardiovascular endurance and practical reasoning. This is in line with studies of class time PA programs in mainstream schools which report positive academic [51], behavioural [50] and physical fitness outcomes [112]. These results also align with suggestions from McMinn, Rowe and Trim [35] who indicate that classroom PA programs may contribute to cognitive and social benefits for students with special needs. However, considerable variance between the included programs (e.g., type of program, session duration, frequency etc.) and a lack of large RCT studies makes it difficult to infer robust conclusions specific to outcomes. Therefore, more, large RCT studies are needed. Additionally, it was beyond the scope of this mapping review to conduct a detailed review of the effects of the programs. Further research is required to explore preliminary indications using statistical approaches such as meta-analysis. Moreover, the findings in this review are descriptive and therefore do not provide an understanding of how the programs impact areas of child development. Nevertheless, this review does provide an overview of existing evidence and suggests that further research is warranted. 

### 4.4. Considerations for Particular Disability Populations

The results of this review highlight the importance of considering disability populations separately when developing and evaluating class time PA programs. Indeed, the most common type of activity implemented with children with disabilities often differed between different disability populations, suggesting that a program appropriate for children with one type of disability may not be the most suitable for all children with special needs. Additionally, the impacts of the programs are likely to vary for children with different disabilities. This is perhaps expected given the heterogeneity of children with disabilities and aligns with a review by Rimmer et al. [113] which outlines the necessity of developing exercise programs for particular disability groups. As noted, given the paucity of research available, the findings from the current review are preliminary and descriptive. Future research should investigate these indications further to provide an understanding of appropriate class time PA programs for children with particular disabilities. It is possible that this information would be useful for classroom teachers in specialist education settings when putting class time PA programs into practice, particularly with regards to providing suggestions of how to select or modify program activities for children with disabilities. It is also likely that future programs will need to be designed in a way that allows flexibility to tailor to the strengths of the students of interest given the variability among students, even those with the same disability type.

### 4.5. Risk of Bias

There is a paucity of high-quality evidence from the evaluations of programs included in this review, with most (n = 17) receiving an overall weak rating. Only three studies produced moderate-strength evidence. None were rated as strong. A number of consistencies in risk of bias and several methodological limitations across the studies may contribute to this. For example, many studies were at risk of selection bias due to not investigating participants that are representative of the target population, or not providing enough information about sample representativeness. Many studies were also at risk of performance and detection bias, as blinding processes were infrequently utilised or clearly reported. Additionally, many studies did not clearly report on or employ intervention integrity processes. Indeed, studies rarely reported the percentage of participants that received the allocated intervention and whether participants were likely to be exposed to contamination or co-intervention, which could have compromised the impact of the program. Furthermore, very few evaluations utilised an RCT design. This is consistent with previous literature reporting few RCTs of PA interventions for people with disabilities [55,113]. Many also consisted of modest sample sizes.

These consistencies in risk of bias may be due to factors such as the study age, study type (i.e., theses), sample heterogeneity and constraints inherent to conducting research in schools. Indeed, several complexities inherent to the specialist education context (e.g., difficulties achieving equal participant groups due to the heterogeneity of students’ disabilities, difficulty recruiting a sufficient number of students and high resource needs) may make robust RCTs with large sample sizes difficult to conduct [97,113]. Understandably, intervention research is lacking in specialist education settings [114] and may require more resourcing to produce robust research findings. Nevertheless, more rigorously designed studies are required to advance the quality of scientific evidence that informs effective practices for class time PA programs in specialist schools.

### 4.6. Strengths and Limitations

This is the first study to the authors’ knowledge that reviews class time PA programs in the specialist education context. The review included a comprehensive and systematic search of peer-reviewed and grey literature sources and dual screening of results. However, this review is subject to limitations. Firstly, it is possible that programs akin or identical to those in this review have been implemented in other contexts without documentation and therefore were not found during the searches. Secondly, limiting search results to English may have neglected programs implemented in non-English speaking countries. Thirdly, it is possible that other identified programs could have been included if authors were contactable for extra information [115,116]. Lastly, although comments were made about program evaluations, it was beyond the scope of this mapping review to explore the effects of the PA programs in detail. Caution should therefore be taken when interpreting results.

## 5. Conclusions

This review has taken the first step towards understanding class time PA opportunities available to children attending specialist schools and has identified a dearth of programs that have been implemented and evaluated. PA programs have been successfully implemented during class time in mainstream schools [45,50] and appear to have the potential for use in specialist education settings [35]. Indeed, this review identified promising indications of a number of developmental benefits that children with disabilities may experience from engaging in a range of physical activities during class time. However, inconsistencies across studies create challenges for interpreting and generalising these indications and signal the need for further investigation. While there are a number of inherent challenges to overcome, the development, implementation and evaluation of additional class time PA programs in specialist schools is warranted. Particularly, more robust studies are required to evaluate class time PA programs in specialist schools in order to develop a high-quality evidence base to inform classroom practice [97]. Given the biopsychosocial health benefits of PA, addressing the identified gaps in class time PA programs in specialist education settings could positively impact the developmental trajectory of children with disabilities.

## Figures and Tables

**Table 1:** Key search terms used to search electronic databases.

Concept	Search String
Participant	((child*) OR (youth) OR (pediatric) OR (paediatric) OR (minors) OR (girls) OR (boys) OR (kid*) OR (student*))
Participant	((disab*) OR (“special needs”) OR (“developmental* challenge*”) OR (impair*) OR (handicap*) OR (“neurodevelopmental disorder*”) OR (retard*) OR (“development* disorder*”) OR (ASD) OR (“Autism Spectrum Disorder*”) OR (autis*) OR (ADHD) OR (“Attention Deficit Hyperactivity Disorder”) OR (“Cerebral Palsy”) OR (“Developmental Coordination Disorder”) OR (Blind) OR (Deaf*) OR (wheelchair) OR (“Down Syndrome”) OR (“Emotion* Behavio* Problem*”) OR (“Fragile X”) OR (Dyspraxia) OR (“Cystic Fibrosis”) OR (“Mental Disorder*”) OR (anxiety))
Setting	((classroom*) OR (class) OR (classes))
Setting	((“special* school*”) OR (“special education school*”) OR (“primary school*”) OR (“elementary school*”) OR (“junior school*”) OR (“infant school*”) OR (“special needs school*”) OR (“special development* school”))
Intervention	((“physical activit*”) OR (exercis*) OR (movement) OR (moving) OR (fitness) OR (“adapted physical education”) OR (“motor activit*”))

Note. Some of the key terms in this table are no longer appropriate to describe children with disabilities. However, given the terms were used to search much earlier literature, it was necessary to include them. * is the symbol used for truncation of search terms.

**Table 2:** Characteristics of included records.

Author (Year)	Country	Study Design	Comparison	Total Sample Size	Participant Sex	Participant Age in Years	Participant Disability Category	Participant Disability Description
Allen (1980) [66]	USA	Post-only	Days without jogging	N = 12	M: 12 F: 0	Range: - *M*: -	Special needs	Behavioural and/or perceptual disorders and limited gross motor skills in a class for children with learning difficulties.
Allen (1989) [67]	USA	Pre-post	Two classes at a different school continued with normal routine (1 h PE every week)	N = 28	M: 15 F: 13	Range: 9–15 *M*: 11.8	Special needs	All had a developmental disability with *M*_IQ_ of 69. The IQ range for the intervention group was 63–74 (*M*_IQ =_ 68.5) and for the comparison group was 59–80 (*M*_IQ =_ 69.5).
Barton (1979) [68]	USA	Pre-post	None	N = 21	M: 13 F: 8	Range: 9–16 *M*: -	ID	All had an ID.
Bass (1985) [69]	USA	Reversal	Non-running days	N = 6	M: 4 F: 2	Range: 8–11 *M*: -	Special needs	All had learning disabilities but did not receive medication.
Bellitto (1981) [70]	USA	Pre-post	None	N = 10 ^a^	M: 7 F: 3	Range: - *M*: -	ID	ID with deficits in gross and fine motor abilities, as well as auditory perception skills.
Bernstein (1985) [71]	USA	Observational	None	N = 48	M: 42 F: 6	Range: 6–9 *M*: -	Special needs	Majority had learning disorders and/or social adjustment difficulties. Many were 2 years behind anticipated age/grade levels.
Bothma, Dunn and Kokot (2014) [72]	South Africa	Pre-post	Comparison group took part in placebo activities	N = 18	M: 10 F: 8	Range: 4.5–8 *M*: -	Sensory impairments	All had severe to profound bilateral hearing loss.
Bruce, Fasy, Gulick, Jones and Pike (2006) [73]	USA	No evaluation reported	None	N = -	M: - F: -	Range: 3–10 *M*: -	Multiple disabilities	Severe and multiple disabilities including developmental delays, autism, physical disabilities and congenital deafblindness.
Carnahan, Musti-Rao and Bailey (2009) [62]	USA	Reversal	Not applicable ^b^	N = 6	M: 5 F: 1	Range: 6–11 *M*: 9 y 1 m	ASD	Autism or “other health impairment”. All were 2–5 years below grade level functioning and had difficulties with academic engagement.
Davis, Zhang and Hodson (2011) [74]	USA	Pre-post	None	N = 25	M: 16 F: 9	Range: 8–12 *M*: 9.7	ID	All had mild to moderate ID.
Dickinson and Place (2016) [75]	UK	RCT	Control group received standard school PE program	N = 67 ^a^	M: 54 F: 13	Range: not reported -11 *M*: -	ASD	ASD and a moderate or severe intellectual difficulty in classes for children with marked ASD.
Doherty (1971) [76]	Canada	Controlled trial	Control group received the usual PE	N = 29	M: 16 F: 13	Range: 8–12 *M*: -	ID	IQ between 49 and 65. Perceptual Motor program group: *M*_IQ =_ 57.3. Physical Conditioning program group: *M*_IQ =_ 55.8. Control group: *M*_IQ =_ 56.7.
Everhart, Dimon, Stone, Desmond and Casilio (2012) [77]	USA	Reversal	Non-intervention periods	N = 13	M: - F: -	Range: - *M*: -	ID	All had an ID.
Fontana and Diaper (1981) [78]	UK	Controlled trial	Control group continued with usual classroom tasks	N = 20	M: 20 F: 0	Range: 7–9 *M*: -	ID	Average IQ was 68.7 and 66.8 for the experimental and control groups respectively. Many did not meet the reading age classification and showed signs of significant maladjustment.
Gitter (1967) [79]	USA	No evaluation reported	None	N = 13	M: 13 F: 0	Range: 8–11 *M*: -	Special needs	IQ ranged from 58 to 72.
Government of WA, Department of Health (2015) [80]	Australia	No evaluation reported	None	N = 33	M: - F: -	Range: - *M*: -	ID	All had an ID.
Hall and Deacon (1970) [65]	USA	Pre-post	Control group engaged in the usual special class curriculum	N = 40 ^a^	M: - F: -	Range: 8–15 *M*: -	ID	All had an ID. Group 1 IQ: 30–53. Group 2 IQ: 30–54.
Halle, Silverman and Regan (1983) [81]	USA	Pre-post	None	N = 9	M: - F: -	Range: 6.25–11.75 *M*: 8.5	ID	All had an ID.
Lu, Petersen, Lacroix and Rousseau (2010) [64]	Canada	Action research	None	N = 25	M: 23 F: 2	Range: 7–12 *M*: 9.9	ASD	All had ASD. Some also had motor delays and impaired hearing.
Miller, Rynders and Schleien (1993) [82]	USA	Controlled trial	Children were allocated to either the drama or cooperative games group	N = 8 ^a^	M: - F: -	Range: - *M*: - ^c^	ID	ID ranged from moderate to profound difficulty.
Ministry of Education, Singapore (2018) [83]	Singapore	No evaluation reported	None	N = -	M: - F: -	Range: - *M*: -	ID	All had an ID.
Nelson, Paul and Barnhill (2017) [84]	USA	No evaluation reported	None	N = -	M: - F: -	Range: 4–7 *M*: -	Sensory impairments	All had significant visual impairments and additional disabilities in a class for children who are blind or vision impaired.
Nicholson (2008) [85]	USA	Single subject multiple baseline	None	N = 4	M: 4 F: 0	Range: 9–9 *M*: -	ASD	All had high-functioning ASD. Two had a diagnosis of Asperger’s and two had a diagnosis of autism.
Nunley (1965) [86]	USA	Pre-post	None	N = 11	M: - F: -	Range: 9–14 *M*: 12.5	ID	All had a moderate ID. IQ ranged from 36 to 55.
Oriel, George, Peckus and Semon (2011) [87]	USA	RCT	Control condition where children did a classroom task without first doing aerobic exercise	N = 9	M: 7 F: 2	Range: 3–6 *M*: 5.2	ASD	All met educational criteria for ASD, seven of which had a formal diagnosis of autism. One had a primary diagnosis of ID and one had a primary diagnosis of developmental delay.
Roswal, Sherrill and Roswal (1988) [88]	USA	Controlled trial	Classes were allocated to either the data-based dance pedagogy or the creative dance pedagogy	N = 35	M: 23 F: 12	Range: 11–16 *M*: -	ID	All had a moderate ID.
School Annual Report (2011) [89]	Australia	No evaluation reported	None	N = -	M: - F: -	Range: - *M*: -	ID	All had a moderate or severe ID.
School Website- Aquatics (n.d) [90]	Australia	No evaluation reported	None	N = -	M: - F: -	Range: - *M*: -	ASD	All had a diagnosis of ASD and many also had an ID, a language disorder and complex behaviours.
School Website- Outdoor Education (n.d) [91]	Australia	No evaluation reported	None	N = -	M: - F: -	Range: - *M*: -	ASD	All had a diagnosis of ASD and many also had an ID, a language disorder and complex behaviours.
School Website- Swimming (n.d) [92]	Australia	No evaluation reported	None	N = -	M: - F: -	Range: - *M*: -	Multiple disabilities	All had ASD and ID.
Seham (2012) [93]	USA	No evaluation reported	None	N = -	M: - F: -	Range: - *M*: -	Multiple disabilities	Severe physical, cognitive and emotional disabilities.
Spanbauer (1990) [94]	USA	No evaluation reported	None	N = -	M: - F: -	Range: - *M*: -	Sensory impairments	All had hearing impairments. There was also a Special Opportunities class for children who had other conditions in addition to deafness.
Taylor (1964) [95]	USA	Pre-post	None	N = 10	M: 0 F: 10	Range: 7–11 *M*: -	Special needs	IQ from 61 to 79 and all were functioning 2 to 4 years behind in school.
Walton (1979) [96]	USA	Pre-post	None	N = 5	M: 5 F: 0	Range: 12–14 *M*: -	Emotional difficulties	All had severe emotional difficulties.

Note. ASD: autism spectrum disorder. F: female. ID: intellectual disability. M: male. *M*: mean. n.d: no date. PE: physical education. RCT: randomised controlled trial. WA: Western Australia. ^a^ Additional participants were described, however they are not included in this review as they do not meet the inclusion criteria. ^b^ The comparison group was related to a different activity in this multicomponent program and is therefore not relevant to this review. ^c^ The mean age reported in this study included children who were typically developing; therefore, the mean age of the students with disabilities specifically is not known.

**Table 3:** Description of included programs.

Author (Year)	Program Category	Program Description	Program Duration	Program Delivered by	Program Location	Outcome Category(ies)	Outcome(s) Evaluated
Allen (1980) [66]	Running	Exercise periods consisting of warm up stretches, 5–10 min of continuous movement (run, jog or walk) and cool down stretches.	A 15 min session, twice per week for 6 weeks.	The classroom teacher	School grounds (on a 1/3 mile track around the school playground)	Behavioural	Classroom behaviour.
Allen (1989) [67]	Dance/drama	A dance/movement program. Lesson themes were based on Laban’s principles. Lessons aimed to increase body awareness, teach self-expression and communication through movement, and develop movement vocabulary. Lessons also aimed to increase social, psychomotor, cognitive and affective skills. Each lesson included a warm up, exploration of the daily theme, movement sequences, demonstration to the class and cool-down.	A 1 h session, every school morning for 2 weeks.	Researchers	School gymnasium (or large space in the school)	Psychological	Self-concept.
Barton (1979) [68]	Dance/drama	An aerobic dance program. Each lesson consisted of a warm-up, sit-ups, work-out and cool-down. The work-out segment (25–30 min) involved learning dance routines with a focus on monitoring the children’s heart rate to dictate intensity level.	A 45 min session, 3 times per week for 8 weeks.	Researchers (a graduate student helped with delivery)	School gymnasium (or ‘all purpose room’)	Physical Psychological	Physical fitness. Self-concept.
Bass (1985) [69]	Running	A running program. Children ran with the whole class on a quarter-mile track.	A 45 min session on alternate mornings for 4 weeks.	Not reported	Other (on a quarter-mile track)	Cognitive Behavioural	Attention span. Impulse control.
Bellitto (1981) [70]	Motor activities Games/play	A gross motor curriculum involving body image, non-locomotor and locomotor tasks. Body image tasks involved identifying body parts and included games such as the Hokey Pokey. Non-locomotor tasks involved activities such as balance, pushing/pulling, twisting and bouncing, and used games such as Simon Says and parachute play. Locomotor tasks involved activities such as running, hopping, lifting and leaping, and included games such as hopscotch and pretending to be animals.	A 45 min session, every school morning for 30 weeks.	The classroom teacher (who was also the researcher)	The classroom	Academic Physical	Academic abilities. Gross motor skills.
Bernstein (1985) [71]	Dance/drama	Spolin theatre games. These activities consist of more than 200 non-competitive group theatre games and exercises that promote social interaction and creativity.	A 45 min session, twice per week for 10 weeks.	Researchers	Not reported	Social	Children’s social relations.
Bothma, Dunn and Kokot (2014) [72]	Motor activities	The Wired to Learn program. This movement program consists of 10 main activities. Children learn and continue to practice the first movement activity until they can do it with ease. Children then proceed through the movement activities in order.	A 15–25 min session ^a^, once per day (5 times per week) for 14 weeks.	Researchers	School room (the school clinic)	Other Physical Social Cognitive	Total developmental age. Locomotor development. Personal-social development. Language development. Eye-hand coordination development. Performance development. Practical reasoning development.
Bruce, Fasy, Gulick, Jones and Pike (2006) [73]	Stretching	Morning Circle meetings. Among many components, stretching and yoga have been included in Morning Circle meetings. The stretching component involved imitating the teacher. The yoga component consisted of students taking turns to select a yoga position and everyone imitating the position depicted.	Yoga components lasted no longer than 10 min. Other stretching activities were less than 10 min.	The classroom teacher	The classroom	No outcomes evaluated	None.
Carnahan, Musti-Rao and Bailey (2009) [62]	Dance/drama	A 30 min class group activity which consists of peer greetings, a calendar activity, a picture book activity and a movement/dancing activity. The movement or dancing time included activities such as the Hokey Pokey and YMCA.	Every school day for 8 weeks. The duration of the movement component specifically is unclear.	The classroom teacher	The classroom	Not applicable	Not applicable.
Davis, Zhang and Hodson (2011) [74]	Motor activities Games/play	The Motivate, Adapt, and Play program. Each 30 min session consisted of a warm up, cardiovascular activities, strength activities and a flexibility/closure activity. Tasks were designed to be fun activities presented in a game-style format that is appropriate and motivating for students with ID.	A 30 min session every school day for 8 weeks.	Classroom teacher 3 times a week. Graduate students twice a week.	Not reported	Physical	BMI. Cardiovascular endurance. Muscular endurance. Arm strength. Flexibility.
Dickinson and Place (2016) [75]	Games/play	Mario & Sonic at the Olympic Games on the Nintendo (Kyoto, Japan) Wii™. Children were able to choose to play athletics, aquatics, fencing or table tennis games electronically using the motion sensor of the Wii console remote.	A 15 min session, 3 times per week for 9 months.	Other (school staff)	School room (where PE would normally take place)	Social Other	Social functioning. Family functioning.
Doherty (1971) [76]	Motor activities Games/play	Two experimental programs; a Perceptual Motor program and a Physical Conditioning program. The Perceptual Motor program included activities such as balancing, throwing, hopping, crawling and obstacle courses. The Physical Conditioning program included activities such as races, tag, tug-of-war, sit ups and jumping. Activities gradually increased in difficulty and each child progressed at their own rate.	A 45 min session, 3 times per week for 5 months.	Researchers (university PE students and housewives assisted)	School gymnasium	Physical Academic Cognitive Other	Physical fitness. Perceptual motor abilities. Sensory integration skills. Academic functioning. Cognitive development.
Everhart, Dimon, Stone, Desmond and Casilio (2012) [77]	Dance/drama Other	Primary students followed a 10 min aerobic dance DVD. Intermediate students followed a 10 min TaeBo DVD.	A 10 min session on 19 days over a 30 day period.	The classroom teacher	Not reported	Academic	Mathematics academic activity. Language arts academic activity.
Fontana and Diaper (1981) [78]	Motor activities	A remedial movement program involving group and individual activities including locomotor, coordination, body awareness, laterality, rhythm and balance tasks.	A 45 min session, twice per week for 12 weeks.	Researchers	Not reported	Cognitive	Psycho-linguistic ability.
Gitter (1967) [79]	Motor activities	A circle or ellipse was drawn in the classroom and children were taught to walk along the line. Difficulty was applied by asking the child to balance objects (e.g., glass of water, a bell or a bean bag) while walking the line.	Not reported.	The classroom teacher	The classroom	No outcomes evaluated	None.
Government of WA, Department of Health (2015) [80]	Motor activities	Daily morning PA sessions. The sessions involve engaging in a set of gross movement skills in line with class therapy plans. The program also focuses on reflexes, with specific goals for each student. Some classes join mainstream groups in various fitness activity stations around the school.	A 15–20 min session ^a^ every school day.	The classroom teacher (sometimes Education Assistants).	Not reported	No outcomes evaluated.	None.
Hall and Deacon (1970) [65]	Motor activities	The Frostig Program for the Development of Visual Perception. This involved 60 min of work sheet activities and 30 min of physical activities.	A 30 min session every school day for 7 months.	Not reported	Not reported	Not applicable	Not applicable.
Halle, Silverman and Regan (1983) [81]	Running	A running program. Children ran a quarter mile track four times per week and a 600 yard track on the fifth day. Goals were set for each student to determine the distance they were required to run and the time they were required to complete it in.	A 15 min session, once per day for 7 months.	The classroom teacher	School grounds (perimeter of the playground or a footpath across school grounds)	Physical Other	Fitness. Program satisfaction.
Lu, Petersen, Lacroix and Rousseau (2010) [64]	Games/play	Sandplay workshops. Each session involved an opening ritual (5–10 min of physical, verbal and imaginary activities including mirroring, naming feelings and play-acting to encourage fine and gross motor movements and rhythm; e.g., pretending to be animals and eat different foods), sandplay, storytelling, and a closing ritual (a period of handclapping and dynamic physical movements).	1 session per week for 10 weeks. The duration of the movement components specifically are not known.	Other (two art therapists)	The classroom	Not applicable	Not applicable.
Miller, Rynders and Schleien (1993) [82]	Dance/drama Games/play	A drama program and a cooperative games program. Each session commenced with a warm up exercise and brief instruction. Activities then took place for approx. 30 min before finishing with a brief discussion. The drama group engaged in theatre games and acting exercises designed by Spolin. The games group participated in non-competitive indoor and outdoor cooperative games.	One approx. 40 min session per week for 3 months.	Other (special education school staff)	Not reported	Social	Initiates positive social interactions. Target of positive social interactions. Quality of friendship.
Ministry of Education, Singapore (2018) [83]	Other	The 1–3-5 fitness program which involves students and teachers exercising together on Mondays, Wednesdays and Fridays ^b^. The Friday exercise includes walking activities (e.g., climbing stairs and walking around the school hall).	A 15–20 min session ^a^ every Friday.	The classroom teacher	School room (hall)	No outcomes evaluated	None.
Nelson, Paul and Barnhill (2017) [84]	Motor activities Dance/drama	A greeting activity based on the BEST (Body, Energy, Space and Time) model that encouraged children to share their name and a body movement with the class. The activity also used rhythm and music to provide dance time and opportunities to repeat body movements.	Not reported.	Other (instructors and classroom aides)	The classroom	No outcomes evaluated	None.
Nicholson (2008) [85]	Running	A 12 min jog followed by five minutes of cool down exercises (i.e., walking and stretching).	A 12 min session, 3 times per week for 5 weeks.	Not reported	School gymnasium	Academic	Academic engaged time.
Nunley (1965) [86]	Motor activities	A PA program involving basic neuromuscular tasks and modified activities from the “Youth Physical Fitness: Elements of a School Centered Program”. Activities were added to target mobilization, strength and coordination and included tasks such as crawling, rolling, hopping, skipping, jumping, push-ups and star-jumps. Activities were changed according to performance improvements or indications of boredom.	A 30–45 min session ^a^ every school day for 15 months.	The classroom teacher (classroom aide also contributed).	School room (auditorium)	Physical Behavioural	Motor abilities. Endurance. Behaviour.
Oriel, George, Peckus and Semon (2011) [87]	Running	A period of running/jogging as a group. If children would not run, jumping on a mini trampoline was provided as an alternative. The session finished with light stretching and a glass of water.	15 min sessions for 3 weeks. Total number of sessions not reported.	Researchers	Not reported	Academic Behavioural	Correct academic responses. Incorrect academic responses. Stereotypic behaviours. On-task behaviour.
Roswal, Sherrill and Roswal (1988) [88]	Dance/drama	Two experimental programs that aimed to teach children 10 skills relevant to dance using different approaches. The Data Based Dance group used a turn-taking approach where the teacher individually taught children the relevant skill while the rest waited for their turn. The Creative Dance group involved the teacher using 15 different lesson plans to teach the dance skills using movement exploration activities and games.	40 lessons over 8 weeks. Each session lasted 30 min.	The classroom teacher (the investigator sometimes assisted)	Not reported	Physical Psychological	Dance skills. Motor performance. Self-concept.
School Annual Report (2011) [89]	Swimming	A swimming program. Children develop their swimming abilities and stroke skills and some also engage in hydrotherapy.	At least 1 session per week for the school year. Sessions ranged from 15 min to 1 h.	Specialist subject teacher (professional swimming instructors)	Not reported	No outcomes evaluated	None.
School Website- Aquatics (n.d) [90]	Swimming	An aquatics program. The program focuses on water safety (e.g., floating skills, water entry skills and rescue techniques) and swimming stroke skills (e.g., freestyle and backstroke).	A 30 min session, once per week for the school year.	Specialist subject teacher (swimming teachers)	Not reported	No outcomes evaluated	None.
School Website- Outdoor education (n.d) [91]	Games/play Other	An outdoor education program. Sessions involve a range of different community engagement activities including learning to share equipment, road safety, playing outdoor games and going on walking excursions.	1 session every week of the school year. Session length varies depending on the class.	The classroom teacher (with a specialist teacher)	Mixed (in the classroom, outdoors and in the community)	No outcomes evaluated	None.
School Website- Swimming (n.d) [92]	Swimming	In-term swimming lessons to develop swimming skills.	A 45 min session every school day for 2 weeks.	Not reported	Not reported	No outcomes evaluated	None.
Seham (2012) [93]	Dance/drama	Dance sessions. Students from a special education class were partnered with typically developing children. Each lesson generally included an introduction game, warm-up stretching, learning and practicing choreography, ‘across the floor dancing’ and a ‘thank-you’.	1, 45–55 min session ^a^ per week for a school year.	Specialist subject teacher (dance teachers with classroom teachers and therapists)	Not reported	No outcomes evaluated ^c^	None.
Spanbauer (1990) [94]	Dance/drama	The ‘Movement Arts’ program, which combines dancing, acting, poetry and signed singing to assist children to learn to express themselves. Children also make connections between movement, emotions and language.	A 52 min session at least once per week for the school year.	Specialist subject teacher (a Movement Arts teacher)	School room (a Movement Arts classroom/ dance studio)	No outcomes evaluated	None.
Taylor (1964) [95]	Dance/drama	A dance program. Each session involved (1) gathering children using music and actions, (2) introduction of the daily activities which included tasks involving musical instruments and props, locomotor movements, games, learning dance routines, imagery movement tasks and improvising dances, for example, (3) an activity requested by students, (4) a relaxation period.	A 30–45 min session ^a^, twice per week for just over 3 months.	Specialist subject teacher (dance teacher)	Mixed (one session in the gymnasium and the other in the classroom)	Cognitive Physical Other	Psycho-linguistic abilities. Sensori-motor skills. Visual perception skills. Auditory discrimination abilities.
Walton (1979) [96]	Stretching	Relaxation training. The sessions consisted of mental, physical (e.g., tensing and relaxing the body) and movement relaxation (e.g., slow-motion exercises). This program was accompanied by one, 20 min bio-feedback training session per week.	A 15 min session, 3 times per week for 16 weeks.	The classroom teacher	The classroom	Behavioural Physical	Inappropriate behaviours. Muscle tension.

Note. BMI: body mass index. ID: intellectual disability. n.d: no date. Not applicable: The evaluation of the program was not related specifically to the PA component and was therefore not included in the results of this review. Multicomponent programs are described in full in the program description column to provide detail around how PA is included in some classes, however only details related to the PA component were used for the results in this review where applicable. PE: physical education. WA: Western Australia. ^a^ The duration of the sessions in this program was averaged for mapping purposes, as the original duration was reported as a range. ^b^ The Monday and Wednesday programs are conducted before school starts and are therefore not described in this review. ^c^ This program has been evaluated as part of a larger, separate study which includes multiple settings that could not be included in this review. Therefore, the outcomes of the program in the Grade 4 special education class included in this review cannot be determined.

## Supplementary Materials

The following are available online at https://www.mdpi.com/1660-4601/16/24/5140/s1, Table S1: List of Google sites searched, Table S2: PsycINFO Search Strategy, File S3: Example Google Search Strategy.

## References

[1] World Health Organization (2011). World Report on Disability.

[2] Australian Bureau of Statistics (2012). Children with a disability. Australian Social Trends.

[3] Australian Institute of Health and Welfare (2004). Children with Disabilities in Australia.

[4] Gadow K.D., Guttmann-Steinmetz S., Rieffe C., DeVincent C.J. (2012). Depression symptoms in boys with autism spectrum disorder and comparison samples. J. Autism Dev. Disord..

[5] World Health Organization (2011). Summary World Report on Disability.

[6] Martin J. (2013). Benefits and barriers to physical activity for individuals with disabilities: A social-relational model of disability perspective. Disabil. Rehabil..

[7] Jung J., Leung W., Schram B.M., Yun J. (2018). Meta-Analysis of Physical Activity Levels in Youth With and Without Disabilities. Adapt Phys. Activ. Q.

[8] Poitras V.J., Gray C.E., Borghese M.M., Carson V., Chaput J.P., Janssen I., Katzmarzyk P.T., Pate R.R., Connor Gorber S., Kho M.E. (2016). Systematic review of the relationships between objectively measured physical activity and health indicators in school-aged children and youth. Appl. Physiol. Nutr. Metab..

[9] Strong W.B., Malina R.M., Blimkie C.J., Daniels S.R., Dishman R.K., Gutin B., Hergenroeder A.C., Must A., Nixon P.A., Pivarnik J.M. (2005). Evidence based physical activity for school-age youth. J. Pediatr..

[10] Johnson C.C. (2009). The benefits of physical activity for youth with developmental disabilities: A systematic review. Am. J. Health Promot..

[11] Murphy N.A., Carbone P.S. (2008). Promoting the participation of children with disabilities in sports, recreation, and physical activities. Pediatrics.

[12] Dahan-Oliel N., Shikako-Thomas K., Majnemer A. (2012). Quality of life and leisure participation in children with neurodevelopmental disabilities: A thematic analysis of the literature. Qual. Life Res..

[13] Wachob D., Lorenzi D.G. (2015). Brief report: Influence of physical activity on sleep quality in children with autism. J. Autism Dev. Disord..

[14] Janssen I., LeBlanc A.G. (2010). Systematic review of the health benefits of physical activity and fitness in school-aged children and youth. Int. J. Behav. Nutr. Phys. Act..

[15] Howells K., Sivaratnam C., May T., Lindor E., McGillivray J., Rinehart N. (2019). Efficacy of Group-Based Organised Physical Activity Participation for Social Outcomes in Children with Autism Spectrum Disorder: A Systematic Review and Meta-analysis. J. Autism Dev. Disord..

[16] World Health Organization (2010). Global Recommendations on Physical Activity for Health.

[17] Fuezeki E., Engeroff T., Banzer W. (2017). Health benefits of light-intensity physical activity: A systematic review of accelerometer data of the National Health and nutrition examination survey (NHANES). Sports Med..

[18] Powell K.E., Paluch A.E., Blair S.N. (2011). Physical activity for health: What kind? How much? How intense? On top of what?. Annu. Rev. Public Health.

[19] Rosenbaum P., Gorter J. (2012). The ‘F-words’ in childhood disability: I swear this is how we should think!. Child Care Health Dev..

[20] Anderson L.S., Heyne L.A. (2010). Physical activity for children and adults with disabilities: An issue of “amplified” importance. Disabil. Health J..

[21] Maiano C. (2011). Prevalence and risk factors of overweight and obesity among children and adolescents with intellectual disabilities. Obes. Rev..

[22] Einarsson I.O., Olafsson A., Hinriksdóttir G., Jóhannsson E., Daly D., Arngrímsson S.A. (2015). Differences in physical activity among youth with and without intellectual disability. Med. Sci. Sports Exerc..

[23] Carlon S.L., Taylor N.F., Dodd K.J., Shields N. (2013). Differences in habitual physical activity levels of young people with cerebral palsy and their typically developing peers: A systematic review. Disabil. Rehabil..

[24] Hinckson E.A., Curtis A. (2013). Measuring physical activity in children and youth living with intellectual disabilities: A systematic review. Res. Dev. Disabil..

[25] Rimmer J.A., Rowland J.L. (2008). Physical activity for youth with disabilities: A critical need in an underserved population. Dev. Neurorehabil..

[26] Jones R.A., Downing K., Rinehart N.J., Barnett L.M., May T., McGillivray J.A., Papadopoulos N.V., Skouteris H., Timperio A., Hinkley T. (2017). Physical activity, sedentary behavior and their correlates in children with autism spectrum disorder: A systematic review. PLoS ONE.

[27] Boddy L.M., Downs S.J., Knowles Z.R., Fairclough S.J. (2015). Physical activity and play behaviours in children and young people with intellectual disabilities: A cross-sectional observational study. Sch. Psychol. Int..

[28] Leung W., Siebert E.A., Yun J. (2017). Measuring physical activity with accelerometers for individuals with intellectual disability: A systematic review. Res. Dev. Disabil..

[29] Downs S.J., Fairclough S.J., Knowles Z.R., Boddy L.M. (2016). Physical activity patterns in youth with intellectual disabilities. Adapt. Phys. Activ. Q.

[30] Wouters M., Evenhuis H.M., Hilgenkamp T.I. (2019). Physical activity levels of children and adolescents with moderate-to-severe intellectual disability. J. Appl. Res. Intell. Disab..

[31] Webster C.A., Russ L., Vazou S., Goh T., Erwin H. (2015). Integrating movement in academic classrooms: Understanding, applying and advancing the knowledge base. Obes. Rev..

[32] Hills A.P., Dengel D.R., Lubans D.R. (2015). Supporting public health priorities: Recommendations for physical education and physical activity promotion in schools. Prog. Cardiovasc. Dis..

[33] Einarsson I.O., Johannsson E., Daly D., Arngrímsson S.Á. (2016). Physical activity during school and after school among youth with and without intellectual disability. Res. Dev. Disabil..

[34] Telama R., Yang X., Viikari J., Välimäki I., Wanne O., Raitakari O. (2005). Physical activity from childhood to adulthood: A 21-year tracking study. Am. J. Prev. Med..

[35] McMinn D., Rowe D.A., Trim V. (2011). Classroom-based physical activity breaks: Potential for use with children with special educational needs. Int. J. Phys. Educ..

[36] Society of Health and Physical Educators (SHAPE) America What is CSPAP?. https://www.shapeamerica.org/cspap/what.aspx.

[37] Sit C., McKenzie T.L., Cerin E., Chow B.C., Huang W.Y., Yu J. (2017). Physical Activity and Sedentary Time among Children with Disabilities at School. Med. Sci. Sports Exerc..

[38] Sit C.H.P., McKenzie T.L., Cerin E., McManus A., Lian J. (2013). Physical activity for children in special school environment. Hong Kong Med. J..

[39] Powell E., Woodfield L.A., Nevill A.A. (2016). Children’s physical activity levels during primary school break times: A quantitative and qualitative research design. Eur. Phy. Educ. Rev..

[40] Erwin H., Beighle A., Carson R.L., Castelli D.M. (2013). Comprehensive school-based physical activity promotion: A review. Quest.

[41] Dinkel D., Schaffer C., Snyder K., Lee J.M. (2017). They just need to move: Teachers’ perception of classroom physical activity breaks. Teach. Teach. Educ..

[42] Goh T.L., Hannon J., Webster C., Podlog L., Newton M. (2016). Effects of a TAKE 10! classroom-based physical activity intervention on third-to fifth-grade children’s on-task behavior. J. Phys. Act. Health.

[43] Lowden K., Powney J., Davidson J., James C. (2001). The Class Moves!® Pilot in Scotland and Wales: An evaluation.

[44] Pangrazi R.P., Beighle A., Vehige T., Vack C. (2003). Impact of Promoting Lifestyle Activity for Youth (PLAY) on children’s physical activity. J. Sch. Health.

[45] Mahar M.T., Murphy S.K., Rowe D.A., Golden J., Shields A.T., Raedeke T.D. (2006). Effects of a classroom-based program on physical activity and on-task behavior. Med. Sci. Sports Exerc..

[46] Liu A., Hu X., Ma G., Cui Z., Pan Y., Chang S., Zhao W., Chen C. (2008). Evaluation of a classroom-based physical activity promoting programme. Obes. Rev..

[47] Smedegaard S. (2016). Move for Well-being in Schools: Implementing physical activity in the Danish Public School. ACHPER Act. Healthy Mag..

[48] Naylor P.J., Nettlefold L., Race D., Hoy C., Ashe M.C., Higgins J.W., McKay H.A. (2015). Implementation of school based physical activity interventions: A systematic review. Prev. Med..

[49] McMullen J., Kulinna P., Cothran D. (2014). Physical activity opportunities during the school day: Classroom teachers’ perceptions of using activity breaks in the classroom. J. Teach. Phys. Educ..

[50] Kibbe D.L., Hackett J., Hurley M., McFarland A., Schubert K.G., Schultz A., Harris S. (2011). Ten Years of TAKE 10!®: Integrating physical activity with academic concepts in elementary school classrooms. Prev. Med..

[51] Watson A., Timperio A., Brown H., Best K., Hesketh K.D. (2017). Effect of classroom-based physical activity interventions on academic and physical activity outcomes: A systematic review and meta-analysis. Int. J. Behav. Nutr. Phys. Act..

[52] Martin R., Murtagh E.M. (2017). Effect of active lessons on physical activity, academic, and health outcomes: A systematic review. Res. Q. Exerc. Sport.

[53] Erwin H., Fedewa A., Beighle A., Ahn S. (2012). A quantitative review of physical activity, health, and learning outcomes associated with classroom-based physical activity interventions. J. Appl. Sch. Psychol..

[54] Bloemen M., Van Wely L., Mollema J., Dallmeijer A., de Groot J. (2017). Evidence for increasing physical activity in children with physical disabilities: A systematic review. Dev. Med. Child Neurol..

[55] Frey G.C., Temple V.A., Stanish H.I. (2017). Interventions to promote physical activity for youth with intellectual disabilities. Salud. Publica. Mex..

[56] McGarty A., Downs S., Melville C., Harris L. (2018). A systematic review and meta-analysis of interventions to increase physical activity in children and adolescents with intellectual disabilities. J. Intellect. Disabil. Res..

[57] Biddle S.J., Fox K., Boutcher S. (2003). Physical Activity and Psychological Well-Being.

[58] Thomas B., Ciliska D., Dobbins M., Micucci S. (2004). A process for systematically reviewing the literature: Providing the research evidence for public health nursing interventions. Worldviews Evid. Based Nurs..

[59] Organisation for Economic Co-Operation and Development (OECD) (2012). CO1.9: Child Disability.

[60] Halladay A.K., Bishop S., Constantino J.N., Daniels A.M., Koenig K., Palmer K., Messinger D., Pelphrey K., Sanders S.J., Singer A.T. (2015). Sex and gender differences in autism spectrum disorder: Summarizing evidence gaps and identifying emerging areas of priority. Mol. Autism.

[61] Singer J.D., Butler J.A., Palfrey J.S., Walker D.K. (1986). Characteristics of special education placements: Findings from probability samples in five metropolitan school districts. J. Spec. Educ..

[62] Carnahan C., Musti-Rao S., Bailey J. (2009). Promoting Active Engagement in Small Group Learning Experiences for Students with Autism and Significant Learning Needs. Educ. Treat. Children.

[63] Moher D., Liberati A., Tetzlaff J., Altman D.G., The PRISMA Group (2009). Preferred reporting items for systematic reviews and meta-analyses: The PRISMA statement. PLoS Med..

[64] Lu L., Petersen F., Lacroix L., Rousseau C. (2010). Stimulating creative play in children with autism through sandplay. Arts Psychother.

[65] Hall S.L., Deacon K.F. (1970). Effects noted from the use of the Frostig training program with trainable retardates. Train. Sch. Bull. (Vinel.).

[66] Allen J.I. (1980). Jogging can modify disruptive behaviors. Teach Except Child.

[67] Allen B.J. (1989). The Effect of Dance/Movement on the Self-Concept of Developmentally Handicapped Fourth and Fifth Grade Students. Ph.D. Thesis.

[68] Barton B. (1979). The Effects of an Aerobic Dance Program on the Self- Concept and the Development of Physical Fitness in Educable Mentally Retarded Children. Master’s Thesis.

[69] Bass C.K. (1985). Running Can Modify Classroom Behavior. J. Learn. Disabil..

[70] Bellitto F.C. (1981). A Correlation between Gross Motor Development and Academic Success for Children Exhibiting Gross Motor Deficiencies upon Entering School. Ph.D. Thesis.

[71] Bernstein B. (1985). Becoming involved: Spolin theater games in classes for the educationally handicapped. Theory Pract..

[72] Bothma J.-M.v.d.M., Dunn M., Kokot S. (2014). The impact of a developmental movement programme on the performance of rural hearing-impaired children on the Griffiths Scales of Mental Development. S. Afr. J. Psychol..

[73] Bruce S., Fasy C., Gulick J., Jones J., Pike E. (2006). Making Morning Circle Meaningful. Teach Except Child Plus.

[74] Davis K., Zhang G., Hodson P. (2011). Promoting health-related fitness for elementary students with intellectual disabilities through a specifically designed activity program. J. Policy Pract. Intellect. Disabil..

[75] Dickinson K., Place M. (2016). The impact of a computer-based activity program on the social functioning of children with autistic spectrum disorder. Games Health J..

[76] Doherty G. (1971). The Effects of Specific Perceptual-Motor Training on the Physical Fitness, Perceptual-Motor Skills, Academic Readiness, and Academic Functioning of Educable Mentally Retarded Children. Ph.D. Thesis.

[77] Everhart B., Dimon C., Stone D., Desmond D., Casilio M. (2012). The influence of daily structured physical activity on academic progress of elementary students with intellectual disabilities. Education.

[78] Fontana D., Diaper R. (1981). Effects of a Special Remedial Movement Programme upon Linguistic Development in ESN (M) Boys. Educ. Psychol. (Lond.).

[79] Gitter L.L. (1967). Montessori principles applied in a class of mentally retarded children. Ment. Retard..

[80] Government of Western Australia (Department of Health) (2015). WA Healthy Schools Project Case Studies 2014.

[81] Halle J.W., Silverman N.A., Regan L. (1983). The effects of a data-based exercise program on physical fitness of retarded children. Educ. Train. Ment. Retard..

[82] Miller H., Rynders J.E., Schleien S.J. (1993). Drama: A medium to enhance social interaction between students with and without mental retardation. Ment. Retard..

[83] Ministry of Education Singapore (2018). Special Education for Exceptional Lives.

[84] Nelson C., Paul K., Barnhill B.A. (2017). Creative Dance-Based Communication Intervention for Children With Multiple Disabilities Including Sensory Impairment. Perspect. ASHA Spec. Interest Groups.

[85] Nicholson H. (2008). The Effects of Antecedent Physical Activity on the Academic Engagement of Children with Autism Spectrum Disorders. Ph.D. Thesis.

[86] Nunley R.L. (1965). A physical fitness program for the mentally retarded in the public schools. Phys. Ther..

[87] Oriel K.N., George C.L., Peckus R., Semon A. (2011). The effects of aerobic exercise on academic engagement in young children with autism spectrum disorder. Pediatr. Phys. Ther..

[88] Roswal P.M., Sherrill C., Roswal G.M. (1988). A comparison of data based and creative dance pedagogies in teaching mentally retarded youth. Adapt. Phys. Activ. Q..

[89] Mary Brooksbank School (2011). Mary Brooksbank School Annual School Report.

[90] Eastern Ranges School Curriculum Specialist: Aquatics. https://easternrangesschool.vic.edu.au/curriculum/.

[91] Eastern Ranges School Curriculum Specialist: Outdoor Education. https://easternrangesschool.vic.edu.au/curriculum/.

[92] South Ballajura Campus Students with Special Needs: Community Access in Education Support. https://southballajuraps.wa.edu.au/students-with-special-needs/.

[93] Seham J., Malley S. (2012). Dance partners: A model of inclusive arts education for children and teens with different abilities. The Intersection of Arts Education and Special Education: Exemplary Programs and Approaches.

[94] Spanbauer P. (1990). Movement Arts: Unlocking the World. Perspect. Educ. Deaf..

[95] Taylor L.N. (1964). Developmental Dance in the Education of the Educable Mentally Handicapped Child. Ph.D. Thesis.

[96] Walton W.T. (1979). The use of a relaxation curriculum and biofeedback training in the classroom to reduce inappropriate behaviors of emotionally handicapped children. Behav. Disord..

[97] Odom S.L., Brantlinger E., Gersten R., Horner R.H., Thompson B., Harris K.R. (2005). Research in special education: Scientific methods and evidence-based practices. Except. Child..

[98] Skoning S.N. (2008). Movement and Dance in the Inclusive Classroom. Teach. Except. Child Plus.

[99] Zitomer M.R. (2016). ‘Dance Makes Me Happy’: Experiences of children with disabilities in elementary school dance education. Res. Danc. Educ..

[100] May T., Chan E.S., Lindor E., McGinley J., Skouteris H., Austin D., McGillivray J., Rinehart N.J. (2019). Physical, cognitive, psychological and social effects of dance in children with disabilities: Systematic review and meta-analysis. Disabil. Rehabil..

[101] Water Safety and Swimming. https://education.qld.gov.au/curriculum/school-curriculum/water-safety-and-swimming.

[102] Swimming and Water Safety Education. https://www.education.vic.gov.au/school/teachers/teachingresources/discipline/physed/Pages/swimmingsafety.aspx.

[103] Interm Swimming. https://www.education.wa.edu.au/interm-swimming.

[104] Downs S.J., Boddy L.M., Knowles Z.R., Fairclough S.J., Stratton G. (2013). Exploring opportunities available and perceived barriers to physical activity engagement in children and young people with Down syndrome. Eur. J. Spec. Needs Educ..

[105] Mayes S.D., Calhoun S.L. (2007). Learning, attention, writing, and processing speed in typical children and children with ADHD, autism, anxiety, depression, and oppositional-defiant disorder. Child Neuropsychol..

[106] Pesce C., Leone L., Motta A., Marchetti R., Tomporowski P.D. (2016). From Efficacy to Effectiveness of a “Whole Child” Initiative of Physical Activity Promotion. Transl. J. Am. Coll. Sports Med..

[107] Han J.C., Lawlor D.A., Kimm S.Y. (2010). Childhood obesity. Lancet.

[108] Barr-Anderson D.J., AuYoung M., Whitt-Glover M.C., Glenn B.A., Yancey A.K. (2011). Integration of short bouts of physical activity into organizational routine: A systematic review of the literature. Am. J. Prev. Med..

[109] Einfeld S.L., Ellis L.A., Emerson E. (2011). Comorbidity of intellectual disability and mental disorder in children and adolescents: A systematic review. J. Intellect. Dev. Disabil..

[110] Biddle S.J., Biddle S.J., Fox K., Boutcher S. (2003). Emotion, mood and physical activity. Physical Activity and Psychological Well-Being.

[111] Biddle S.J., Asare M. (2011). Physical activity and mental health in children and adolescents: A review of reviews. Br. J. Sports Med..

[112] Katz D.L., Cushman D., Reynolds J., Njike V., Treu J.A., Katz C., Walker J., Smith E. (2010). Peer reviewed: Putting physical activity where it fits in the school day: Preliminary results of the ABC (Activity Bursts in the Classroom) for fitness program. Prev. Chronic Dis..

[113] Rimmer J.H., Chen M.-D., McCubbin J.A., Drum C., Peterson J. (2010). Exercise intervention research on persons with disabilities: What we know and where we need to go. Am. J. Phys. Med. Rehabil..

[114] Makel M.C., Plucker J.A., Freeman J., Lombardi A., Simonsen B., Coyne M. (2016). Replication of special education research: Necessary but far too rare. Remedial. Spec. Educ..

[115] Oliver J.N. (1958). The effect of physical conditioning exercises and activities on the mental characteristics of educationally sub-normal boys. Br. J. Educ. Psychol..

[116] Chasey W.C., Wyrick W. (1971). Effects of a physical developmental program on psychomotor ability of retarded children. Am. J. Ment. Defic..

