# Supplementary material for: Class Time Physical Activity Programs for Primary School Aged Children at Specialist Schools: A Systematic Mapping Review
Source: Int J Environ Res Public Health. 2019 Dec 16;16(24):5140. doi: 10.3390/ijerph16245140 (PMC6950186; doi:10.3390/ijerph16245140)
Supplement: Supplementary file 1 [file ijerph-16-05140-s001.zip › File S3.docx]

**Supplementary File 3.** Example Google Search Strategy

(children OR students) classroom (“primary school” OR “elementary school” OR “special school” OR “special education”) (“physical activity” OR movement OR exercise OR “motor activity”) (disability OR challenge OR “special needs” OR impairment) site:vic.gov.au
