# Supplementary material for: Class Time Physical Activity Programs for Primary School Aged Children at Specialist Schools: A Systematic Mapping Review
Source: Int J Environ Res Public Health. 2019 Dec 16;16(24):5140. doi: 10.3390/ijerph16245140 (PMC6950186; doi:10.3390/ijerph16245140)
Supplement: Supplementary file 1 [file ijerph-16-05140-s001.zip › Table S1-proof.docx]

**Supplementary Table 1.** List of Google sites searched.

| **Site type** | **Google search operator** |
| --- | --- |
| Australian federal health | site:health.gov.au, site:healthpromotion.org.au |
| Australian state government | site:vic.gov.au, site:tas.gov.au, site:qld.gov.au, site:nsw.gov.au, site:sa.gov.au, site:wa.gov.au, site:nt.gov.au, site:act.gov.au |
| Australian state education | site:vic.edu.au, site:tas.edu.au, site:qld.edu.au, site:nsw.edu.au, site:sa.edu.au, site:wa.edu.au, site:nt.edu.au, site:act.edu.au |
| WHO | site:who.int |
| UNICEF | site:unicef.org |
| UNESCO | site:unesco.org |
| Canada education | site:edu.gov.on.ca |
| UK education | site:gov.uk |
| USA education | site:ed.gov |
| Japan education | site:mext.go.jp |
| New Zealand education | site:education.govt.nz |
| Singapore education | site:moe.gov.sg |
| South Africa education | site:gov.za |
