# Supplementary material for: Class Time Physical Activity Programs for Primary School Aged Children at Specialist Schools: A Systematic Mapping Review
Source: Int J Environ Res Public Health. 2019 Dec 16;16(24):5140. doi: 10.3390/ijerph16245140 (PMC6950186; doi:10.3390/ijerph16245140)
Supplement: Supplementary file 1 [file ijerph-16-05140-s001.zip › Table S2.docx]

**Supplementary Table 2.** PsycINFO Search Strategy

| **Database** | **Search Strategy** | **Limits** |
| --- | --- | --- |
| PsycINFO via EBSCO | ( TI child* OR AB child* OR TI youth OR AB youth OR TI pediatric OR AB pediatric OR DE "Pediatrics" OR TI paediatric OR AB paediatric OR TI minors OR AB minors OR TI girls OR AB girls OR TI boys OR AB boys OR TI kid* OR AB kid* OR TI student* OR AB student* OR DE "Students" )  AND  ( TI classroom* OR AB classroom* OR DE "Classrooms" OR TI class OR AB class OR TI classes OR AB classes )  AND  ( TI “primary school*” OR AB “primary school*” OR DE "Primary School Students" OR TI “elementary school*” OR AB “elementary school*” OR DE "Elementary Schools" OR DE "Elementary School Students" OR TI “junior school*” OR AB “junior school*” OR TI “infant school*” OR AB “infant school*” OR TI “special* school*” OR AB “special* school*” OR TI “special education school*” OR AB “special education school*” OR DE "Special Education" OR TI “special needs school*” OR AB “special needs school*” OR TI “special development* school” OR AB “special development* school” )  AND  ( TI “physical activit*” OR AB “physical activit*” OR DE "Physical Activity" OR TI exercis* OR AB exercis* OR DE "Exercise" OR TI movement OR AB movement OR DE "Movement Therapy" OR TI moving OR AB moving OR TI fitness OR AB fitness OR DE "Physical Fitness" OR TI “motor activit*” OR AB “motor activit*” OR TI “adapted physical education” OR AB “adapted physical education” )  AND  ( TI disab* OR AB disab* OR DE "Disabilities" OR DE "Developmental Disabilities" OR DE "Learning Disabilities" OR DE "Multiple Disabilities" OR TI “special needs” OR AB “special needs” OR DE "Special Needs" OR DE "Special Education Students" OR TI “developmental* challenge*” OR AB “developmental* challenge*” OR TI impair* OR AB impair* OR TI handicap* OR AB handicap* OR TI “neurodevelopmental disorder*” OR AB “neurodevelopmental disorder*” OR DE "Neurodevelopmental Disorders" OR TI retard* OR AB retard* OR DE "Cognitive Impairment" OR DE "Intellectual Development Disorder" OR TI “development* disorder*” OR AB “development* disorder*” OR TI ASD OR AB ASD OR TI “Autism Spectrum Disorder*” OR AB “Autism Spectrum Disorder*” OR DE "Autism Spectrum Disorders" OR TI autis* OR AB autis* OR TI ADHD OR AB ADHD OR DE "Attention Deficit Disorder with Hyperactivity" OR TI “Attention Deficit Hyperactivity Disorder” OR AB “Attention Deficit Hyperactivity Disorder” OR TI “Cerebral Palsy” OR AB “Cerebral Palsy” OR DE "Cerebral Palsy" OR TI “Developmental Coordination Disorder” OR AB “Developmental Coordination Disorder” OR DE "Dyspraxia" OR TI Blind OR AB Blind OR DE "Vision Disorders" OR DE "Blind" OR TI Deaf* OR AB Deaf* OR DE "Hearing Disorders" OR DE "Deaf" OR DE "Partially Hearing Impaired" OR TI wheelchair OR AB wheelchair OR TI “Down Syndrome” OR AB “Down Syndrome” OR DE "Down's Syndrome" OR TI “Emotion* Behavio* Problem*” OR AB “Emotion* Behavio* Problem*” OR TI “Fragile X” OR AB “Fragile X” OR DE "Fragile X Syndrome" OR TI Dyspraxia OR AB Dyspraxia OR TI “Cystic Fibrosis” OR AB “Cystic Fibrosis” OR DE "Cystic Fibrosis" OR TI “Mental Disorder*” OR AB “Mental Disorder*” OR DE "Mental Disorders" OR TI anxiety OR AB anxiety OR DE "Anxiety Disorders" ) | English |
